# Putative dopamine neurons in the ventral tegmental area enhance information coding in the prefrontal cortex

**DOI:** 10.1038/s41598-018-29979-2

**Published:** 2018-08-06

**Authors:** Camilo J. Mininni, César F. Caiafa, B. Silvano Zanutto, Kuei Y. Tseng, Sergio E. Lew

**Affiliations:** 1 Instituto de Biología y Medicina Experimental (IBYME), CONICET, Buenos Aires, Argentina; 2 Instituto Argentino de Radioastronomía (IAR) - CCT La Plata, CONICET - CICPBA, Villa Elisa, Argentina; 3 Universidad de Buenos Aires, Facultad de Ingeniería, Instituto de Ingeniería Biomédica, Buenos Aires, Argentina; 4 Department of Anatomy and Cell Biology, College of Medicine, University of Illinois at Chicago, Chicago, IL 60612 USA

## Abstract

It has been proposed that neuronal populations in the prefrontal cortex (PFC) robustly encode task-relevant information through an interplay with the ventral tegmental area (VTA). Yet, the precise computation underlying such functional interaction remains elusive. Here, we conducted simultaneous recordings of single-unit activity in PFC and VTA of rats performing a GO/NoGO task. We found that mutual information between stimuli and neural activity increases in the PFC as soon as stimuli are presented. Notably, it is the activity of putative dopamine neurons in the VTA that contributes critically to enhance information coding in the PFC. The higher the activity of these VTA neurons, the better the conditioned stimuli are encoded in the PFC.

## Introduction

The prefrontal cortex (PFC) and the ventral tegmental area (VTA) are key brain regions within the neural circuit of reward. Firstly, the PFC is involved in several cognitive functions, including working memory^1^, behavioural inhibition^2^, and learning^3,4^. In addition, considerable evidence has led to propose that PFC neurons code reward-related cues by means of increments in their firing rate^5–7^. Secondly, dopamine neurons in the VTA project to the PFC^8,9^, playing a critical role in the regulation of cognitive function and motivational state. For example, excessive activation or blockade of dopamine receptors in the PFC impairs performance in working memory (WM) tasks^10,11^. Moreover, D1 and D2 agonists enhance stimuli coding in PFC neurons^12,13^, while D1 antagonists impair coding and learning of novel stimulus-response pairings^3,14^. These findings are in line with modelling studies showing that different mechanisms of action by dopamine in the PFC are required to achieve stable neural representations^15–18^. Therefore, it has been proposed that dopamine levels set PFC dynamics into a suitable state for proper coding of relevant stimuli^19^. In a recent study (Mininni *et al*.^20^), we found that the coding capacity in PFC increases efficiently during stimuli presentation in a Go/NoGO discrimination task. Further theoretical considerations suggest that changes in PFC signal-to-noise correlation ratio induced by VTA dopamine neurons could explain our results. Yet, current understanding of the dynamics underlying information coding between the PFC and VTA remains incomplete. While this issue can be addressed through simultaneous neurophysiological recordings, few studies have adopted this experimental approach. Among these, recordings in anaesthetized animals have shown negative correlations between VTA Local Field Potential (LFP) and membrane potential of PFC pyramidal neurons^21^, and between VTA firing rate and PFC LFP^22^. In behaving rats, PFC neurons are phase-locked to VTA oscillations, together with an increased PFC-VTA coherence during the decision period of a WM task^23^.

In the present study, we conducted simultaneous recordings of neuronal activity in the PFC and VTA from trained rats employed in Mininni *et al*. to determine how PFC coding of conditioned stimuli is modulated by stimulus-evoked activity of putative dopaminergic (pDA) and non-dopaminergic (Non-DA) neurons in the VTA. We found that mutual information between stimuli and PFC neuronal activity increases during stimuli presentation in a manner that is proportional to the magnitude of pDA neuronal response.

## Results

**Figure 1:**
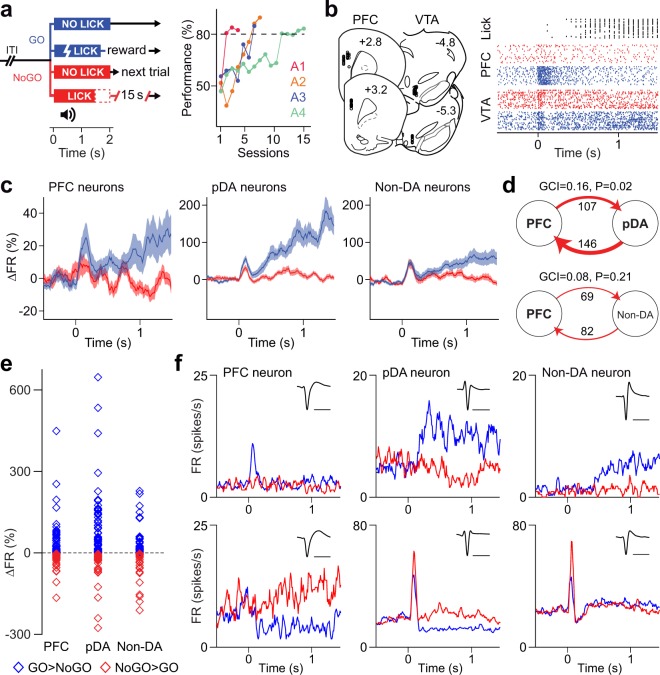
Patterns of neuronal response to GO/NoGO cues in the PFC and VTA. (**a**) Summary of the GO/NoGO paradigm used. A random 1–3 s pre-stimulus delay was followed by a 1 s tone (1 KHz or 8 KHz), after which the licking response was measured during a 2 s opportunity window. Only correct GO responses were rewarded with a drop of water, while correct NoGO trials were followed by a shorter inter-trial interval (ITI), giving the chance to get water sooner in subsequent trials. Four out of six trained animals achieved the criterion of session performance higher than 80% (dashed line) with a NoGO performance higher than 60%. **(b)** Left: recording sites for all PFC - VTA electrode placements relative from bregma. Each dot represents the electrode position for a specific recording session. Right: Raster plots illustrating examples of licking responses (in black) and spiking activity of a PFC neuron and a VTA neuron during 30 consecutive correct GO trials (blue) and NoGO trials (red). Note the increment in firing rate immediately after presentation of the tone. **(c)** Relative to baseline values (mean +/− s.e.m., 100 ms bins), PFC and VTA neurons increased their mean firing rate (%ΔFR) following the presentation of GO and NoGO cues. However, this increment was significantly greater during GO trials in both PFC (n = 95, p < 0.01, GO vs. NoGO, Sign test) and pDA (n = 94, p < 0.0001, GO vs. NoGO, Sign test) neurons. In contrast, Non-DA cells exhibited similar patterns of response during GO and NoGO trials (n = 59, p = 0.6, GO vs. NoGO, Sign test). **(d)** Granger-causality analysis of the mean firing rate revealed an asymmetric directionality from the VTA to the PFC. Only the GCI for the pDA group is statistically different from the expected GCI under the hypothesis of no temporal structure. This suggests that pDA neurons (not Non-DA neurons) are likely to Granger-cause PFC cell firing during tone presentation. The numbers next to the arrows indicate the n of pairs of PFC-VTA neurons for each interaction. **(e)** Dot plot showing PFC, pDA and Non-DA neuronal responses (ΔFR relatively to baseline) during GO and NoGO trials. In blue are neurons exhibiting higher firing rate during GO trials whereas in red are neurons responding with higher firing rate during NoGO trials. **(f)** Examples of peri-stimulus time histograms (10 ms bins) illustrating the pattern of neuronal responses from the GO > NoGO (top) and the NoGO > GO (bottom) groups. Scale bar: 1 ms.

Rats were first trained to perform an auditory GO/NoGO discrimination task using a head-fixed paradigm, with 4 out of 6 rats reaching criteria (Fig. 1a). Thus, all the subsequent analyses were assessed from these 4 rats during task performance. We analysed 30 simultaneous recordings, encompassing 95 neurons in PFC and 153 neurons in VTA, see Supplementary Table S1 and Fig. 1b. Cell activity in the VTA was grouped into two clusters (pDA vs. Non-DA) according to the Euclidean distance between their waveforms and the waveforms of pramipexole responding neurons recorded from anesthetised rats (Supplementary Fig. S2). Figure 1b shows typical raster plots of PFC and pDA neurons, together with the licking responses executed during 30 consecutive correct GO responses. PFC and VTA neurons responded to the presentation of the stimulus by an increment in their mean firing rate, as revealed by the percentage of change computed for correct trials only (Fig. 1c). Both pDA and Non-DA neurons increased their firing rate during GO stimuli, with a more prominent increase for pDA neurons (p < 0.0001, comparing the firing rate changes of pDA and Non-DA neurons at +0.1 s, Sign test). During the tone, PFC neurons also fired more for GO tone than NoGO tone, regardless of tone frequency (see Methods: Discrimination Task Training, ΔFR_GO1KHz_ = 0.139, ΔFR_NoGO8KHz_ = 0.023, p < 0.01, Wilcoxon Rank Sum test; ΔFR_GO8KHz_ = 0.37, ΔFR_NoGO1KHz_ = −0.047, p < 0.05, Wilcoxon Rank Sum test).

To assess whether a directional relationship between VTA and PFC exists, we measured Granger Causality^24,25^ among the across trials average activity of all VTA-PFC pairs of neurons. For the group of pDA neurons, we found 146 pairs where VTA Granger-cause PFC (p < 0.01, F test) while for the group of Non-DA neurons we found 82 pairs where VTA Granger-cause PFC (p < 0.01, F test). Since we also found PFC neurons that Granger-cause VTA neurons, we computed a causality index (GCI, see *Methods*) that varies between −1 (all PFC neurons significantly Granger-cause VTA neurons) and 1 (all VTA neurons significantly Granger-cause PFC neurons). The GCI allows us to condense GC values for both interaction directions (PFC → VTA or VTA → PFC) into a single measure for which a distribution under the null hypothesis of no directionality can be constructed. To assess the significance of GCI values, we built 500 surrogates of neuronal pairs, by shuffling the order of time bins, such that all temporal structure is disrupted (see *Methods*). GCI for pDA neurons was 0.16 (p < 0.02) while GCI for Non-DA neurons was 0.086 (p = 0.21), see Fig. 1d. Not all PFC and VTA neurons responded in the same way to stimulus presentation. A summary of each neuronal preference for GO and NoGO trials is shown in Fig. 1e, together with examples of peri-stimulus time histograms (Fig. 1f).

**Figure 2:**
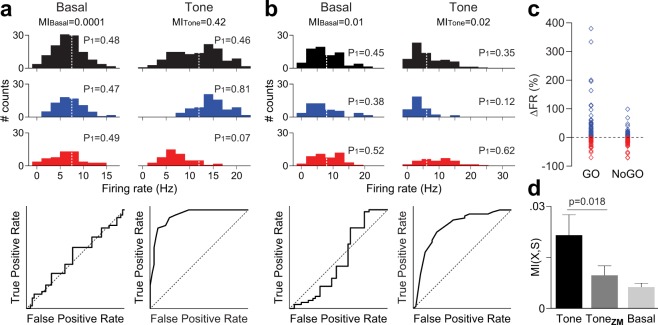
Changes in neuronal firing rate account for information coding in the PFC. **(a)** Histograms summarizing the across-trial firing rate distributions for all (black), GO-only (blue) and NoGO-only (red) trials from a neuron that increased its firing rate during the GO tone and decreased it during the NoGO tone. A 1 s duration window was used to compute the histograms before tone presentation (Basal) and during the tone (Tone). White dashed lines indicate the mean firing rate for all trials. Note that the firing rate distributions for GO and NoGO trials are similar during the basal period (i.e., no information regarding stimulus identity can be extracted). However, the opposite/differential response during tone presentation for both GO and NoGO trials leads to an increased decoding power, as reflected by the ROC curve analysis (AUC = 0.93). The information conveyed by the neuronal response is also evidenced by the marked increase in mutual information (MI) value computed from the binary model (the probability P1 of being in a high state is shown for each condition). **(b)** Summary of the across-trial firing rate histograms from a neuron with opposite firing behaviour to that shown in **a**. Here, the neuron codes stimuli by decreasing its firing rate during GO tone and increasing it during the NoGO tone, leading to an increased decoding power during tone presentation as revealed by the ROC curve analysis (AUC = 0.84). **(c)** Dot plot showing PFC neuronal responses (ΔFR relative to baseline) exhibiting increased (blue) or decreased (red) firing rate during GO and NoGO trials. **(d)** Average mutual information (MI) for all PFC neurons computed from the binary model during tone presentation. Average MI decreases when mean firing rate values are subtracted within GO and NoGO conditions (MI_ZM_), becoming indistinguishable from basal values. This suggests that PFC neurons are coding stimuli by means of increments or decrements in their average firing rates.

Although neurons in the PFC changed their firing rate in different directions when the auditory stimuli were presented, the contribution of these changes could be additive in terms of information. Figure 2a,b show an example where the firing rate of two different PFC neurons was shifted after the presentation of a GO and a NoGO stimulus. These neurons changed their firing rate in opposite directions, both of them were reliably able to discriminate the stimulus, as indicated by its Receiver Operating Characteristic (ROC) curve. Within the PFC, neuronal activity tends to increase more frequently during GO trials (68 out of 95 neurons) and to decrease during NoGO trials (55 out of 95 neurons) (Fig. 2c). In fact, PFC neurons were able to decode the auditory stimulus within a wide range of decoding performances, as indicated by the area under the ROC curves.

Given that PFC neurons may code information by either increasing or decreasing their firing rate, we employed mutual information (MI) as a measure of information content in PFC to estimate the amount of information conveyed by neural activity regardless of the direction of firing rate changes. To this end, we first built a two-state neuron model that allowed a reliable estimation of MI^20^. In the model, we set the output of every neuron to ‘0’ or ‘1’ depending on whether the number of spikes within a given time window was lower/higher than the average computed in the same window across all correct GO and NoGO trials. For the two examples given in Fig. 2a,b, MI computed under the binary model increased dramatically during tone presentation.

Information about stimuli can be coded in several statistical characteristics of the distribution of neural activity, i.e., its mean value, its variance or its skewness, among other higher order statistics. We reasoned that if changes in mean firing rate could account for all the information conveyed by PFC neurons, subtracting the mean firing rate from the set of GO and NoGO trials would leave no information remaining. By doing this operation we show that the zero-mean dataset has significantly less information about stimulus when compared with the non-subtracted firing rates (Fig. 2d, MI_ToneZM_ Vs. MI_Tone_, n = 95, p < 0.05, Sign test). Moreover, MI for subtracted firing rates are not statistically different from MI of basal firing rates (Fig. 2d, MI_ToneZM_ Vs. MI_Basal_, n = 95, p = *0*.*37*, Sign test), suggesting that all the information conveyed by PFC neurons during tone presentation is coded as increments or decrements of their firing rate.

**Figure 3:**
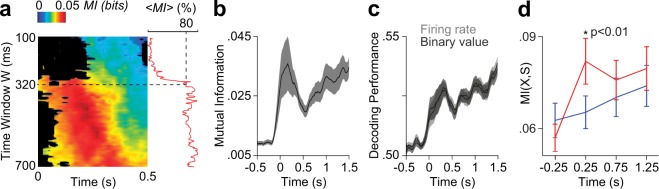
Mutual information analysis of PFC neural response. **(a)** MI between stimuli and pairs of PFC neurons depends on the size of the window of analysis and the time from stimulus onset. Each point in the heat map represents the averaged MI value across PFC neuron pairs for a given window size and a given time point measured from stimulus onset. MI values not significantly different than basal values (p > 0.05, Sign test) are displayed in black. Note that the averaged-across-time MI values (red line, <*MI*>) grows fast as the window size approaches 320 ms (dashed line), reaching 80% of the maximum <*MI*> computed across all window sizes. Further increments in window size does not result in higher <*MI*> values, for which W = 320 ms was chosen as the window size in subsequent analysis. **(b)** MI along the trial, computed in windows of 320 ms. After the onset of the auditory stimuli, MI reaches a peak at 250 ms and remains above chance from then on (mean ± s.e.m. is shown). **(c)** Linear decoders were trained to predict the tone identity (GO or NoGO) from the firing rate of pairs of PFC neurons or from their corresponding binary values. The plot shows the average classification performance computed across all pairs of PFC neurons. Note that performance for firing rates and binary values highly overlaps, suggesting that the binary model retains all information contained in the firing rate extractable by the linear decoders. Mean +/− s.e.m. of 100 bootstrapping iterations are shown. **(d)** Mutual information was higher for trials preceded by different tones (GO vs. NoGO, red line) than for trials preceded by the same stimulus (NoGO, blue line; *p < 0.01, Sign test). This implies that information conveyed by pairs of neurons in the PFC during tone presentation is primarily driven by stimulus-related events rather than the behavioural response itself (lick or no-lick).

To obtain the best temporal resolution constrained to a reliable measure of MI, we looked for the shortest window that maximized MI between stimuli and pairs of neurons. We found that MI peaked shortly after stimulus onset for a time window of 320 ms (n = 159 PFC neuron pairs, p < 0.001, Sign test comparing MI at t = 100 ms and t = −500 ms), accounting for 80% of the maximum MI value (Fig. 3a). Therefore, pairs of neurons and a time window of 320 ms were selected for subsequent analysis. In Fig. 3b, we show the dynamic of mutual information between stimuli and pairs of neurons for a sliding window (320 ms) along the trial. We then asked to what extent our simplified binary model was losing information contained in the actual firing rate. We used a Fisher linear discriminant (FLD) trained with either binary states or firing rates, and computed the classification performance along stimulus presentation (Fig. 3c). We found no significant differences in discrimination performance, proving that the binary model employed retains all the information that is extractable by the FLD.

Despite the above results showing PFC neurons conveying information associated to the stimuli, it is also possible that the motor response preparation could contribute to MI values as well. For instance, neuronal activity could be coding the action of licking rather than the GO stimulus. To test this possibility, we computed MI between neural activity and stimulus identity over trials where only GO responses were executed and compared it with the MI between neural activity and the response executed over trials were only NoGO tones were presented. Note that MI constrained to trials in which the licking action was the behavioural response (Fig. 3d, red line) was significantly greater than MI between neural activity and behavioural response constrained to NoGO trials only. Together, these results indicate that MI in the PFC is predominantly driven by stimulus-related events.

We hypothesized that the activity of pDA neurons in the VTA modulates the information conveyed by PFC neurons about the stimuli. More specifically, we proposed that the information content in PFC is proportional to the firing rate of VTA-DA neurons, in such a way that the higher their average activity the higher the information content in PFC.

**Figure 4:**
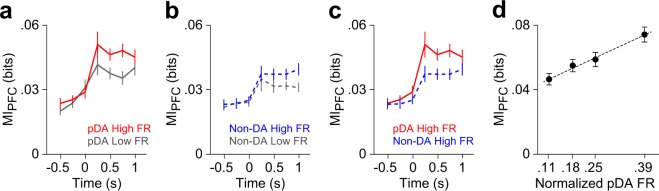
VTA-driven modulation of information in the PFC. **(a,b)** MI between pairs of PFC neurons and stimulus were computed for trials with high and low VTA activity, based on the mean firing rate (FR) of pDA and non-DA neurons measured during the duration of tone presentation. MI in the PFC is higher for trials with high FR than with low FR activity of pDA neurons (**a**; n = 159 pairs, p < 0.05, Sign test). In contrast, PFC MI values are similar across trials with high and low FR of Non-DA neurons (**b**; n = 159 pairs, p = 0.3, Sign test). **(c)** MI analyses for trials limited to high FR VTA activity revealed that PFC MI values are maximized by pDA rather than by non-DA neurons (n = 159 pairs of neuron, p < 0.05, Sign test at +0.5 s). **(d)** MI values in the PFC increased proportionally to the normalized FR activity of pDA neurons (MI computed at +0.5 s, ρ = 0.2, p < 1.10^−6^, Pearson correlation, N = 151 pairs × 4 quarters; dashed line shows linear least square fitting). In all panels mean ± s.e.m. is shown.

To determine the contribution of pDA and Non-DA neurons’ activity on the coding capacity of PFC, we sorted trials according to the normalized firing rate of VTA neurons across trials (pDA and Non-DA neurons), splitting them into a high activity group H (trials where the normalized activity of VTA was above the mean across trials), and a low activity group L (trials where the normalized activity of VTA was below the mean across trials). Results show that when H and L groups were built based on the activity of pDA neurons, MI in the PFC is higher for H than for L groups (n = 159 PFC neuron pairs, p = 0.002, 2-way repeated measures ANOVA; Fig. 4a), and no differences were observed when the ordering corresponds to the activity of Non-DA neurons (n = 159 PFC neuron pairs, p = 0.20, 2-way repeated measures ANOVA; Fig. 4b) nor when H and L groups were selected based on the firing rate of PFC neurons and MI was computed over pDA neuron pairs (n = 173 PFC neuron pairs, p = 0.24, 2-way repeated measures ANOVA, Supplementary Fig. S3). Moreover, MI in the PFC is higher for a pDA-obeying ordering than a Non-DA one within H groups (p = 0.03 bootstrapped analysis, 200 repetitions; Fig. 4c), showing that MI in the PFC is more strongly related to the firing rate of pDA than non-DA neurons. Notably, MI in the PFC grows monotonically when trials were sorted in groups of increasing global activity of pDA neurons (Fig. 4d).

To gain insight into the mechanisms underlying MI differences, we investigated how the average firing rate and its variability changed across trials when GO and NoGO stimuli were presented. Thus, we measured the signal-to-noise ratio (SNR) of PFC neurons during tone presentation, as the inverse of the firing rate coefficient of variation, see *Methods*. In the case of pDA-based definition of H and L, the presentation of GO tones leaded to higher SNR values for the H group than for the L group (SNR_H-GO_ = 0.178, SNR_L-GO_ = 0.132, p = 0.012, Sign test), while no differences were observed for the NoGO tones (SNR_H-NoGO_ = 0.020, SNR_L-NoGO_ = 0.011, p = 0.063, Sign test) or when the H and L groups were defined based on a non-DA ordering (SNR_H-GO_ = 0.141, SNR_L-GO_ = 0.138, p = 0.73, Sign test; SNR_H-NoGO_ = −0.0042, SNR_L-NoGO_ = 0.0113, p = 1, Sign test). It is worth noting that none of the constructed sets of trials had a systematic bias for GO or NoGO trials (p > 0.25, Sign Test comparing the proportion between the number of GO and NoGO trials, across sessions for each condition: High/Low, pDA/non-DA). This fact excludes any possibility of an effect due to trial type misbalance.

Altogether, these results suggest that fluctuations in the amount of stimuli–related information conveyed by PFC neurons are better explained by the trial by trial activity of pDA rather than non-DA VTA neurons.

## Discussion

Modulation of PFC activity by dopamine is critical for a variety of cognitive functions such as working memory, decision making and goal directed behaviour^26^. Yet, the precise contribution of dopamine activity in shaping and sustaining PFC computation remains unclear. Here we found that conditioned stimuli elicited pDA neuronal responses were associated with higher coding capacity in the PFC, as determined by mutual information analyses of simultaneously recorded PFC-VTA activity. Mutual information (MI) is a non-parametric measure that assesses the amount of information that is shared between two stochastic variables. It makes no assumptions about the underlying neural code and allows a better estimate of information in highly variable processes such as in neural populations. We combined MI computations with surrogate trial sets built according to VTA population activity and found that PFC neurons conveyed more information in response to conditioned stimuli (CS) during trials in which the firing rate of pDA neurons was higher than their average firing rate across trials. In fact, PFC-CS MI increased monotonically with the firing rate of pDA neurons. Previous work have shown that dopamine agonists injected into the PFC can change the coding capacity of PFC neurons^13^. Similarly, repeated optogenetic stimulation of VTA-DA projections to the PFC can potentiate the response of PFC neurons to paired stimuli and facilitates learning^27^. However, both pharmacological and optogenetic manipulations were delivered continuously over tens of trials, precluding the analysis of short-term modulations. In this regard, our present study was designed to fill this gap in knowledge by computing the activity of ongoing PFC-VTA interactions and determine the dynamics of PFC coding capacity in a trial by trial basis. Our data revealed that the coding capacity in the PFC can increase from trial to trial, depending on the degree of pDA neuronal activation in the VTA.

Braver and Cohen (2000) proposed that PFC function is to maintain task-relevant information through a dopamine-dependent manner. According to this model, coordinated activation of the dopamine system is required to enable proper neuronal ensembles in the PFC to selectively amplify salient information. Our simultaneous recordings present a scenario that is consistent with this theory: pDA neurons increment their firing rate along with GO stimulus and PFC neurons are more prone to increase their firing rate with GO rather than NoGO trials, regardless of perceptual features, i.e. tone frequency. This could mean that pDA neurons become more responsive to a stimulus that requires an action. Moreover, we found that encoding of relevant stimuli by PFC neurons increases with higher pDA neuronal activity, while higher PFC neuronal activity does not lead to better stimuli coding in the VTA. To further test that the VTA–PFC interaction was asymmetric, we applied the GCI analysis using a surrogate dataset in which time bins were shuffled such that all temporal structure were disrupted. Together, our findings revealed a model in which pDA neuronal activity modulates PFC neuronal response in order to improve coding of task-relevant information. Further studies are warranted to determine to what extent the impact of pDA neuronal activity on PFC coding is causal.

The mechanisms underlying the sustained increments in firing rate of PFC neurons as a coding strategy have been studied in the context of attractor dynamics^15–18^. In this framework, dopamine projections from the VTA modulate the excitability of both excitatory and inhibitory neurons in the PFC through a variety of mechanisms, which in turn act synergistically towards greater stability of the activated representations. For example, the NMDA-D1 synergism and the action of fast spiking interneurons (FSI) could be responsible for improving PFC selectivity. The first mechanism stresses that coordinated activation of glutamate NMDA and dopamine D1 receptors in the PFC^28,29^ is required to increase the signal detection ratio of afferent information. On the other hand, dopamine has been found to increase the firing rate of inhibitory interneurons in the PFC, limiting the response of pyramidal neurons^30,31^ and reducing irrelevant background activity^32^. Thus, the deepening in the basins of attraction can be shown by measuring the signal-to-noise ratio in the firing rate of PFC neurons. Previous works in PFC-VTA co-cultures^33^ and in anaesthetized rats^34^ revealed that dopamine can increase the signal-to-noise ratio of PFC neurons by reducing the level of spontaneous firing and enhancing afferent-evoked responses. Here, we showed in awake behaving rats that the signal-to-noise ratio of PFC neurons was also higher when pDA neurons fired above their mean firing rate during GO tone presentation. Together, these results indicate that dopamine-mediated increments in PFC coding of task relevant stimuli may occur through a fine-tuning of signal-to-noise ratio in the PFC.

In summary, our work shows how pDA neuronal activity impacts PFC output coding. Even though our training protocol does not enable the omission of expected rewards nor the variation of reward magnitude, we observed that the coding capacity in the PFC fluctuates with the firing rate of pDA neurons. In this regard, other studies have shown that tonic changes in dopamine levels affect the motivational state of the animal^35^. To what extent the PFC coding capacity can be influenced by differences between fast and slow dopamine-driven modulation remains to be determined.

## Materials and Methods

All experimental procedures involving animals were approved by the Committee of Animal Care and Ethics from the Instituto de Biología y Medicina Experimental-Consejo Nacional de Investigaciones Científicas y Técnicas (IByME-CONICET) in accordance with the National Institute of Health Guide for Care and Use of Laboratory Animals. Some of the experimental and analytical procedures have been described previously. See Mininni *et al*.^20^ for reference.

### Animals and pre-surgical handling

Adult (2-month-old) male Long Evans rats (N = 6) were obtained from the IByME-CONICET vivarium, housed individually with food and water *ad libitum*, and kept on a 12/12 h dark/light cycle. In order to habituate animals to movement restriction they were handled during two weeks before surgery, lifting them for a short time firstly (30 s) that was increased up to 10 min.

### Head fixation device

Fixation devices were cross-shaped aluminium pieces (2 gram) manufactured from 2 mm thick aluminium sheet. The four ends of the device were screwed to two plastic adapters, which in turn were fastened to the ear bar holders of a Kopf stereotaxic apparatus, see Supplementary Fig. S4.

### Surgery

Animals were anesthetized using Ketamine/Xylazine (75 mg/kg, 10 mg/Kg, respectively). The proper state of anaesthesia was tested by observing absence of the paw reflex. Throughout surgery, the eyes were covered with ointment to prevent drying out. Body temperature was measured by a rectal probe and held constant at 37 °C using a controlled pad.

The head fur was shaved and the skull was cleaned and disinfected. Once the skull was exposed, two holes of 2 mm in diameter were drilled over the PFC and VTA areas (PFC coordinates: AP= +2.7 mm, L = 0.5 mm, DV = 3 mm, VTA coordinates: AP = −4.8 mm, L = 1 mm, DV = 8 mm; Bregma as zero^36^). A 3 mm diameter by 4 mm deep plastic cylindrical recording chamber was positioned around each hole. Two Stainless-steel screws were positioned in each of the parietal bones (4 screws). Finally, the fixation device was held in place and anchored to the screws and recording chambers with dental acrylic, and a steel pin was attached in order to reference electrode position against Bregma across sessions. The recording chambers were filled with antibiotic solution (Neomycin 3.5 mg/ml, Polymyxin B 5000 UI, Gramicidin USP 0.025 mg; OFTAL 3, Holliday – Scott, AR), and sealed with a cotton cap.

Immediately after surgery rats were subcutaneously injected with 1 mg/kg of the analgesic Meloxicam (Mobic, Boehringer Ingelheim, AR). During postoperative, rats were treated with antibiotic (Enrofloxacin in drinking water at 0.05 mg/ml; Floxacin, Afford, AR) and analgesic (3 drops of Tramadol 5% per 100 ml of drinking water; Calmador, Finadiet, AR) for at least 5 days.

### Electrodes and Data Acquisition

Extracellular recordings were obtained using wired tetrodes^37^. Briefly, they were built from 4 coiled wires of Nychrome of 12 μm in diameter (Kantal, Palm Coast). Each tetrode was then introduced inside a stainless steel cannula of 230 μm of external diameter. Each wire was isolated by a polyamide sheath, and its impedance (at 1 KHz) was adjusted between 0.5 to 0.8 MΩ by gold electro-deposition at the tip. Electrode bundles were built using three cannulae with cyanoacrylate in a triangle configuration and a separation of 250 μm. A wire attached to the cannulae of each set of tetrodes was used to ground the recording system. Signals were pre-amplified x10 and amplified x1000. Data were acquired with a National Instruments device at a sampling frequency of 30 KHz.

### Habituation to head fixation

Seven days after surgery, water supply was progressively reduced down to 12 ml per day, taking care of the animal weight which was never less than 85% of their *ad libitum* weight. To habituate animals to the head fixation framework they were progressively fixed 10, 20, 40, 80, 160 minutes per day to the stereotactic frame while drops of water were delivered sporadically. Animals were kept fixated unless they presented signals of stress such as agitation or teeth chattering. During fixation, the animal body was placed in a half-cylinder bed (7 cm in diameter and 20 cm long) made of PVC.

### Preparatory training

On the first day of training, animals were trained on a single classical conditioning session, where a tone (T1) lasting 1 s was followed by a drop of water (0.06 ml) as reward. On the second day, an operant conditioning protocol was conducted: the same tone was followed by a 2 s window of opportunity to lick in order to get a drop of water. Once the subjects performed above 80% of correct trials in the operant protocol, the discrimination task training was begun.

### Discrimination task training

Rats were trained to learn an auditory discrimination task, under the GO/NoGO paradigm. Each trial started with a random 1-to-3 s delay, followed by a 1-s long stimulus presentation, chosen at random from two possible frequencies (T1: lick tone, and T2: no lick tone). After the tone, the animal had a two seconds opportunity window to execute the response: to protrude the tongue, or to hold it. When the T1 tone was presented and the animal made a lick action, a drop of water was delivered (GO correct trial). There was no reward if the animal did not lick (GO incorrect trial). In the case of GO trials the inter-trial interval (ITI) was 4 s. If after the T2 tone animals withdraw the tongue, no reward was delivered, but the ITI was cancelled and the next trial started immediately (NoGO correct trial). Conversely, a lick action (NoGO incorrect trial) meant no reward and a time out of 15 s as punishment.

In three subjects chosen at random T1 was a 1 KHz tone and T2 an 8 KHz tone, while in the other subjects frequencies were exchanged. Four out of six trained animals reached a performance criterion of 80% of correct trials with at least 60% of correct NoGO trials, and were then passed to recording sessions.

Before a recording session, cotton caps were removed and a few drops of lidocaine 2% were applied on the meninges. Next, the meninges were cut using a 30 gauge needle with the aid of a surgical microscope. Three tetrodes were lowered in each area. When spikes were found in at least one tetrode of each area the behavioural protocol was started. All recording sessions ended when the subject was no longer willing to perform the task.

### Tetrode recordings in anesthetized rats

Two Long Evans rats were anaesthetized with urethane (1.4 g/Kg) and placed in a stereotaxic frame. After exposing the skull, a hole was drilled above the VTA using the same stereotaxic coordinates described above. Three tetrodes were lowered and recordings began when activity in multiple channels was detected. After 10 minutes of stable activity, pramipexole (1 mg/kg, IP) was injected^38,39^. Activity changes were then observed for at least 10 minutes after injection. We tagged neurons as putative dopamine ones when significant decrease in firing rate occurred after pramipexole injection.

### Histology

At the end of the last recording session, rats were deeply anesthetized, electrodes position were electrically marked and then animals were perfused with formalin 4%. The brains were removed and coronal sections containing the PFC and VTA were collected to determine the exact location of the recording electrodes through cressyl violet staining of the sections. Electrode positions in previous sessions were estimated based on their recording coordinates, the reference pin (see Surgery section) and the electric mark of last session, see Fig. 1b.

### Data analysis

#### Spike detection and clustering

Electrophysiological raw data was band filtered (300 Hz to 3 KHz) before spike detection and sorting. The filtered signal was compared with threshold value determined as in^40^:

1 $$Thr=4\sigma \,$$

2 $$\sigma =median\frac{|x|}{0.6745}\,$$

and every time the threshold was surpassed 32 samples around the crossing point were stored as a putative spike. Putative spikes were processed with *Wave_Clus* clustering software^41^.

A preliminary automatic sorting was performed in each channel, followed by manual adjustment of the clusters. Waveforms were aligned to the most prominent peak and its Signal to Noise Ratio (SNR) was computed (as the ratio between the average peak and the signal standard deviation 500 μs before the peak). Based on the stability of both, principal components (PC) and firing rates along the recording, together with the SNR values (SNR > 4)^42^, units were selected as single neurons for the rest of the analysis, see Supplementary Fig. S5.

We constructed rasters at 1 ms time resolution employing the time-stamp of each spike of each isolated unit. Peristimulus time histograms (PSTHs) were constructed by counting spikes occurring within bins of 100 ms length, aligned to stimulus onset. For each neuron we computed the percentage of change in firing rate, by taking the difference between the firing rate at a given time *t* and the basal firing rate defined at −500 ms and dividing the difference by the basal firing rate.

All analyses were conducted on correct trials only.

#### Assessment of VTA-PFC interaction directionality

Neuron activity of VTA and PFC was averaged across trials in non-overlapped bins of 50 ms length in order to build a temporal series for each neuron. Then, Granger Causality (GC^25^,) between VTA and PFC neurons was computed from −1s to 1s around tone onset. We defined a Granger Causality Index (GCI) as follows:

3 $$GCI=\frac{{P}_{VTA\to PFC}-{P}_{PFC\to VTA}}{{P}_{VTA\to PFC}+{P}_{PFC\to VTA}}$$

where ${P}_{X\to Y}$ is the number of *X-Y* pairs for which *X* significantly Granger-causes *Y* (p < 0.01).

To assess GCI significance we built 500 surrogates by shuffling bins in both, PFC and VTA neuronal activity and building a GCIull distribution. We computed the probability p of measuring a bigger GCI value than the value measured from the data, and rejected the null hypothesis if p < 0.05.

#### The binary random model for neurons activity

To study how much information is contained in the neuron population we built a binary neuron model that allows reliable estimation of pairwise Shannon entropy and mutual information^20^. The state of each neuron can be ‘1’ or ‘0’, depending on whether the number of spikes in a given time window was higher or lower than its average across all correct (GO and NoGO) trials. For extremely short windows, our approach is similar to the one employed by other authors^43,44^. There, the probability of finding a neuron in a ‘1’ state is directly related to the existence of a spike inside the window and, in consequence, with its firing rate.

We define the binary random variable ${X}^{i}(t$) associated with neuron $i$ which takes the value ${X}^{i}(t)=1$ if its spike count is greater than the mean across trials in the analysis window *W* centred at time $t$, otherwise ${X}^{i}(t)=0$, being $t=0$ the time of stimulus (tone) onset. For pairs of neurons, ${X}^{ij}(t)$ adopts one out of four possible values: $\{11,10,01,00\}.$

#### Mutual information

We computed Mutual Information $I(X,S)\,$between $X\,$(the state of single neurons or pair of neurons in the binary model) and $S$ (GO and NoGO) at time *t* as follows:

4 $$I(X,S,t)=\sum _{\{X,S\}}P(X,S,t)\,lo{g}_{2}\frac{P(X,S,t)}{P(X,t)P(S,t)}$$

where $\{X,S\}$ is the set of all combinations of binary neural states values and input stimuli measured in a window centred at *t*. Mutual Information bias was computed by shuffling the GO and NoGO labels 30 times, thus obtaining 30 different shuffled data sets, and averaging their Mutual Information values. Due to the fact that the computed bias remains practically constant along the trial (0.018 ± 0.0006, mean and s.e.m.), we worked with raw MI values.

In order to determine the length of the analysis window *W*, we computed Mutual Information $I({X}^{ij},S)\,$for window sizes ranging from *W* = *100* *ms to W* = *700* *ms* centred at a time *t*, which varied from *t* = *0* *ms to t* = *500* *ms*. We computed one MI value for each pair of PFC neurons at a given time *t* and window *W* and averaged the MI values across all pairs. Then we computed$\, < MI >$, the average mutual information across all pairs of neurons and time points for each time window *W* and determined the shortest window that provided a mutual information value of at least 80% of the highest $< MI >$. This length (320 ms) was used for subsequent analysis; see the red curve in Fig. 3a.

To know to what extent the binary model could affect our information measures, we compared the discrimination power of a linear decoder when it was fed with binary states (0/1) or with the firing rates directly. For each pair of neurons, we compute the Fisher Linear Discriminant vector $w$ using 80% of the trials randomly selected as follows:

5 $$w=({\mu }_{GO}-{\mu }_{NOGO})\ast {(\frac{1}{2}{{\rm{\Sigma }}}_{GO}+\frac{1}{2}{{\rm{\Sigma }}}_{NOGO})}^{-1}$$

being ${\mu }_{GO}$ and ${\mu }_{NOGO}$ mean values of activity for the GO and NoGO groups of trials, and ${{\rm{\Sigma }}}_{GO}$ and ${{\rm{\Sigma }}}_{NOGO}$ their covariance matrices. We tested the performance of the decoder with the remaining 20% of the trials. We repeated this analysis 100 times and then estimated the average performance and its bootstrapping standard error to the mean.

#### The role of VTA as a modulator of mutual information in the PFC

We assumed that there was a causal relationship between the average firing rate of VTA neurons and the activity in PFC, so that the spike count distributions observed in the latter are determined by the former.

More specifically, we hypothesized that this VTA-driven modulation impacted on MI in the PFC, in such a way that the higher the average activity of VTA the higher the MI in PFC. To test this hypothesis we normalised the activity of each VTA neuron between the minimum and maximum firing rate across trials, during the whole tone. Then, we averaged trial by trial the normalised activity among VTA neurons (referred from now on as *global* VTA activity of the trial). Trials were sorted in ascending order according to their global VTA activity and divided into two halves: low (L) and high (H). Next, PFC trials were grouped according to their membership to H or L, and MI between the activity of PFC pairs and stimuli was computed for each group. In order to test statistical differences between groups, we performed a bootstrapping analysis. We measure MI in 16 overlapped windows (length 320 ms) along the tone and computed the averaged MI difference between L and H groups across all windows and pairs of PFC neurons. This value was then compared with a null distribution generated by shuffling high (H) and low (L) labels. To analyse whether trials with increasingly higher/lower firing rates of pDA neurons were associated with higher/lower MI in the PFC, we followed the same procedure, grouping trials in four quarters and computing MI for each of these groups. Thus, a MI value was computed for each pair of PFC neurons in all quarters. Statistical significance was assessed by computing Pearson correlation between these MI values and their associated normalised VTA firing rate. In all these analysis, there were no significant differences in the GO/NoGO trials ratio for H and L groups, allowing a reliable estimation of MI.

Signal-to-noise ratio was computed as the inverse of the coefficient of variation of neurons firing rate during tone presentation (1 second window):

6 $$SNR=\frac{{\mu }_{FR}}{{\sigma }_{FR}}$$

where ${\mu }_{FR}$ and ${\sigma }_{FR}$ are the mean and standard deviation of the neuron firing rate, respectively.

#### Statistics

Plots show the mean ± s.e.m. unless otherwise stated. The Sign test and the Wilcoxon rank sum test where employed for statistical comparisons. All comparisons were two-tailed. No a priori statistical tests were run to predetermine sample size.

## Electronic supplementary material

Supplementary information
