# Supplementary material for: Putative dopamine neurons in the ventral tegmental area enhance information coding in the prefrontal cortex
Source: Sci Rep. 2018 Aug 6;8:11740. doi: 10.1038/s41598-018-29979-2 (PMC6079091; doi:10.1038/s41598-018-29979-2)
Supplement: Supplementary file 1 — Supplementary information [file 41598_2018_29979_MOESM1_ESM.docx]

**Supplementary Information**

**Putative dopamine neurons in the ventral tegmental area enhance information coding in the prefrontal cortex**

Camilo J. Mininni ^a^, César F. Caiafa ^b^, B. Silvano Zanutto ^a,c^, Kuei Y. Tseng ^d,*^, Sergio E. Lew ^c,*^

^a^ Instituto de Biología y Medicina Experimental (IBYME), CONICET, Buenos Aires, Argentina

^b^ Instituto Argentino de Radioastronomía (IAR) - CCT La Plata, CONICET

^c^ Universidad de Buenos Aires, Facultad de Ingeniería, Instituto de Ingeniería Biomédica, Buenos Aires, Argentina

^d^ Department of Anatomy and Cell Biology, College of Medicine, University of Illinois at Chicago, Chicago IL 60612

**Supplementary Table S1**. Number of neurons recorded sorted by recording number, brain area and animal.


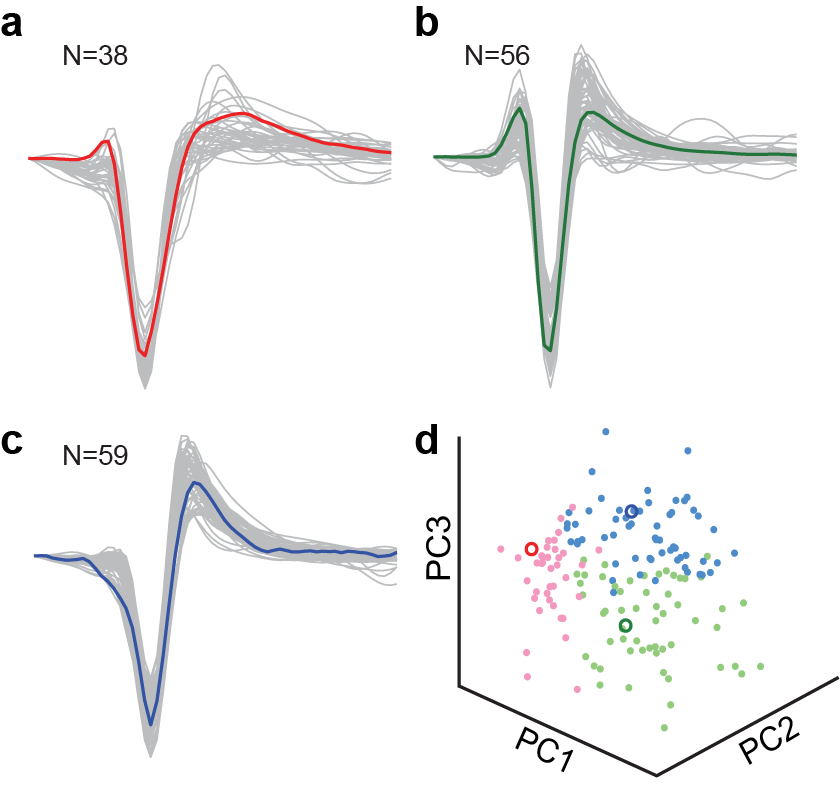
**Supplementary Fig S2. Identification of pDA and Non-DA neurons.** Average spike waveforms of VTA neurons recorded in behaving animals (grey lines) were clustered against the templates (coloured lines) obtained from pramipexole responding and non-responding neurons in anesthetised animals. **(a,b)** Pramipexole responding neurons**.** Two typical waveforms were found, a long lasting one (red in **a**) and a triphasic one (green in **b**). **(c)** A pramipexole non-responding neuron in blue. **(d)** Principal component (PC) analysis of spike waveforms. In filled red, green and blue circles are shown the first 3 PCs for the waveforms recorded in behaving animals. Empty circles show the 3 PCs of the pramipexole responding (red and green) and non-responding (blue) neurons.

**Supplementary Fig. S3. Absence of effect of PFC firing rate on pDA stimuli coding.** MI between pairs of pDA neurons and stimulus were computed for trials with high and low PFC firing rate measured during tone presentation. There were no differences in pDA MI between high and low groups.


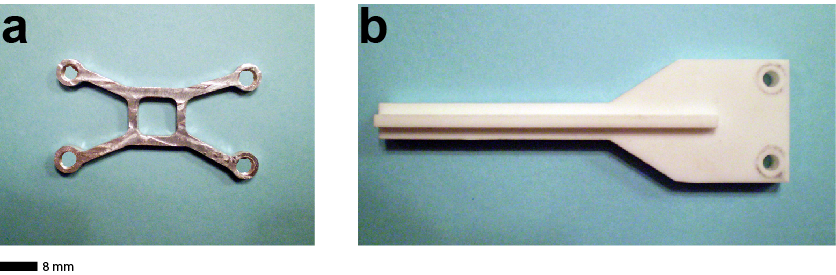
**Supplementary Fig. S4. Fixation device**. **(a)** Aluminium cross-shaped piece (2 gram) manufactured with a 3D Roland MDX 20 milling machine from a 2 mm thick aluminium sheet. **(b)** Plastic adapter that were screwed on both sides of the fixation device and in turn were fastened to the ear bar holders of a Kopf stereotaxic apparatus.


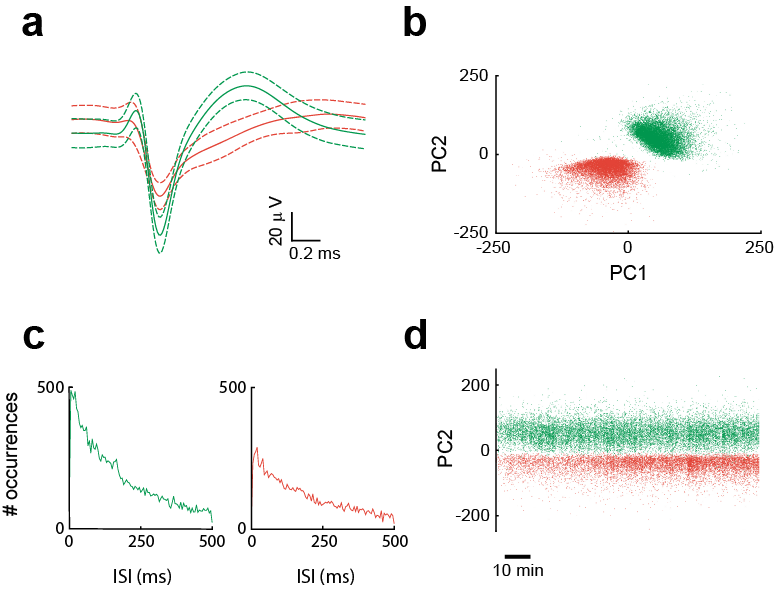
**Supplementary Fig. S5. Spike sorting.** **(a)** Spike waveforms of two units sorted from the same electrode of one tetrode inserted in PFC. Mean (solid line) and standard deviation (dashed line) are plotted. **(b)** First two principal components separate waveforms into two clusters. **(c)** Inter-spike interval (ISI) distribution. There are less than 1% of ISIs bellow 3 ms. **(d)** Second principal component for the two clusters plotted against time. The two clusters can be recognized all along the 110-minutes recording.
